# Out-of-Pocket Healthcare Expenditures in Dependent Older Adults: Results From an Economic Evaluation Study in Mexico

**DOI:** 10.3389/fpubh.2020.00329

**Published:** 2020-07-24

**Authors:** Aarón Salinas-Rodríguez, Betty Manrique-Espinoza, Irina Torres Mussot, Julio Cesar Montañez-Hernández

**Affiliations:** ^1^Center for Surveys and Evaluation, National Institute of Public Health, Cuernavaca, Mexico; ^2^Center for Health Systems Research, National Institute of Public Health, Cuernavaca, Mexico

**Keywords:** dependent older adults, OOP healthcare expenditures, two-part regression model, basic activities of daily living, instrumental activities of daily living

## Abstract

**Background:** Dependence is a significant health-related condition for older adults (OA) and implies that self-care is transferred to other people, the community or institutions. Recent studies have analyzed the relationship between out-of-pocket (OOP) healthcare expenditures and dependence. Nonetheless, these studies were not specifically designed to estimate the economic impact of dependence. Our aim was to estimate the total adjusted annual OOP healthcare expenditures in dependent older adults compared to independent ones. Additionally, we explore the potential combined effect of basic activities of daily living (ADL) and instrumental activities of daily living (IADL) dependence on OOP healthcare expenditures.

**Methods:** Data comes from the cross-sectional study “Economic impact of physical dependence in older adults and the burden of informal care” conducted in 2018 with a sample of 735 community-dwelling older Mexican adults ages 60 and older. We used direct (medical and non-medical) and indirect costs to estimate the OOP healthcare expenditures associated with dependence. We applied the Katz scale to assess dependence in ADL and the Lawton scale to assess dependence in IADL. Two-Part regression models were used to analyze the relationship between dependence and OOP health expenditures.

**Results:** Presence of ADL dependence represented a higher level of expenditure, 107% compared to non-dependent OA (β = 1.07, CI95%: 0.43–1.71), and 97% for IADL dependence (β = 0.97, CI95%: 0.49–1.45). The combined effect of ADL and IADL dependence (132%) was greater (β = 1.32, CI95%: 0.74–1.90) than the effect of ADL or IADL dependence alone. In monetary terms, OA with ADL dependence had a total annualized mean OOP healthcare expenditure of $31,865 (Mexican pesos), OA with IADL $26,912, and combined ADL and IADL $39,520.

**Conclusions:** ADL and IADL dependence are associated with the total annualized OOP healthcare expenditures. This association is even higher when both conditions are present together. These findings highlight the economic implications of the dependence for individuals, their families, and the health system. Given that current evidence on effective interventions to prevent dependence in OA is insufficient, future studies should be conducted to estimate their costs and determine what interventions work, as well as their effectiveness and cost-effectiveness in different sub-groups of the population, and how these might be appropriately implemented.

## Introduction

Dependence is a significant health-related condition for older adults (OA), and its presence implies that self-care is transferred to other people, the community or institutions (1). The World Health Organization (WHO) defines dependence as an event that occurs when the functional or intrinsic capacity has decreased to a point where the person is no longer able to carry out the basic tasks of daily life on their own (2). Dependence in OA is a complex and multidimensional concept that goes beyond a single physical component, encompassing a psychological, and socioeconomic dimension (3). Even so, the dimension of dependence that has been most analyzed in gerontological literature is the physical or functional dependence, and particularly that focused on the functional loss of bodily or sensory abilities (4).

The prevalence of dependence for OA populations in high-income countries ranges from 15 to 17%, while in low and middle-income countries it ranges from 3 to 16%. In Mexico, by 2012, 21.7% of OA had basic activities of daily living (ADL) limitations, and 5.1% were dependents (5).

Dependence has a variety of consequences at different levels. At an individual level, dependence can generate feelings of depression, low self-esteem, and hopelessness (6). At the level of family, it can generate a cycle of impoverishment and reallocation of care roles (7), while at the societal level, dependence can increase demand for health and care services (8). This increase demand for health and care services can also increase the costs associated with healthcare of dependent older people.

The aging process in Mexico (similar to other low- and middle-income countries) has occurred amid a fragile economy marked by high levels of poverty and limited access to health services and resources (9). In that context dependency in old age is one of most critical challenges facing by the social health protection system. Given this scenario, the Mexican Health System, a mixture of public-private schemes, has sought to achieve universal access to health, as well as universal coverage of health services, through three public insurance schemes mainly: The Mexican Institute of Social Security (IMSS by its Spanish acronym), the Institute of Security and Social Services of State Workers [ISSSTE (9)], and by the *Seguro Popular*, today converted into the Institute of Health for Well-being [INSABI (9)]. Despite this, none of these schemes specifically covers the potential health-related costs of physically dependent OA.

In 2012, 13% of older adults (60 years or older) did not have health insurance and 53.5% did not have an economic pension (10). However, since 2009 Mexico has a non-contributory pension program (formerly known as *70 y más*−70 and over, and now as the *Programa para el Bienestar de los Adultos Mayores*-Program for the Welfare of Older Adults) which consists of the bimonthly payment of 2,750 Mexican pesos (115 US dollars approx.), and is intended to be universal to all individuals 68 years of age or older (11, 12).

Recent studies have analyzed the relationship between out-of-pocket (OOP) healthcare expenditures and different health outcomes related to the functional capacity of the OA, namely frailty (13), multimorbidity (14), and disability (15). Some other studies have also examined the relationship between dependence and OOP healthcare expenditures (16–19). Yet, other studies have treated functional dependence as a covariate, rather than the main exposure of interest (20–22). Despite this evidence, some gaps remain, because findings on dependence and healthcare costs have been produced from studies that were not explicitly designed to estimate the economic impact of dependence in older adults.

Also, the combined effects of basic activities of daily living (ADL) and instrumental activities of daily living (IADL) dependence on OOP healthcare expenditures has not been explored. We have designed a study that expressly seeks to better understand the economic implications of dependence in the elderly. Our main objective was to estimate the total adjusted annual OOP healthcare expenditures in dependent older adults compared to independent ones. Additionally, we explored a potential combined effect of ADL and IADL dependence on OOP healthcare expenditures.

## Materials and Methods

### Study Design and Sample

Data comes from the cross-sectional study “Economic impact of physical dependence in older adults and the burden of informal care” conducted in 2018 with a sample of community-dwelling older Mexican adults age 60 and older (23). According to a previous study in which the costs associated with frailty among older Mexican adults were estimated (13), a sample size of 735 OA would have sufficient power to detect significant differences in healthcare costs when comparing individuals with and without dependence based on a significance level of 5%, power of 90%, and one-sided tests. OA in this study were residents of two urban and three rural communities in the Mexican state of Estado de México, located at the central part of Mexico. We defined the analytical sample according to the following criteria: we included all older adults aged ≥60 and were excluded those with hearing or speech impairment. Also, a key informant (proxy) was considered if the OA reported severe memory problems (present if the answer was yes for the question: Have you had difficulties with your memory that are a problem for you?). In that case, the caregiver provided the information (9.7% of the sample). The final analytical sample was comprised of 735 older adults.

We administered an extensive questionnaire to study participants to collect data on the expenditures related to medications, consultations, hospitalizations, laboratory studies, devices, prostheses, and loss of labor productivity. Direct (medical and non-medical) and indirect costs were both used to estimate the economic burden of dependence.

## Measures

### Out-of-pocket Healthcare Expenditures

We calculated total healthcare expenditures based on the estimation of cumulative costs found through the questionnaires. All costs were annualized for 2018. We grouped data in direct and indirect costs as described below.

#### Direct Costs

Medical and non-medical expenditures were estimated using a micro-costing analysis, which allows monetary valuing of the individual use of health resources and services by study participants. Medical costs included medical consultations, specialist consultations, medications (including vitamins), laboratory tests, and hospitalization. Non-medical costs included nursing services, devices (visual, hearing, orthopedic), prostheses, and other costs (personal hygiene, healing material, traditional medicine).

#### Indirect Costs

We operationalized the indirect costs considering the monthly loss of productivity for the OA and his/her carer/relative as follows:

- Loss of labor productivity (OA). We determined the economic loss of labor productivity in working OA by asking about the number of days of work they had missed due to health-related reasons.
- Loss of labor productivity in the household (caregiver or relative). We quantified the loss of labor productivity at the household level by asking relatives how much they thought they had lost on income due to caring for the OA.

### Dependence

We applied two widely known instruments to evaluate the functional capacity of the OA: the Katz and Lawton scales. The Katz scale (24) assess difficulties in ADL, which include bathing, dressing, using the toilet, incontinence, and feeding. The Lawton scale (25) assess difficulties in IADL, including using the phone, going shopping, managing drugs and money, and using public or private transportation.

Based on the information about ADL and IADL, an OA was considered dependent if: (1) they were unable to perform at least one of the ADL or IADL, (2) he/she had a primary caregiver who provided assistance for ADL or IADL, and accompanied him/her to health facilities. Using these criteria, we generated two variables that define either ADL or IADL dependence combining the difficulties in performing ADL or IADL and the presence of a caregiver. These were operationalized as binary variables, where 0 = independent, and 1 = dependent.

### Covariates

We included factors associated with healthcare expenditures in the literature (26, 27) as potential covariates and categorized them as follows:

#### Socio-Demographic Characteristics

Sex (female = 1), age, health insurance (yes = 1), retirement pension (yes = 1), schooling (years of formal instruction), paid job (yes = 1), marital status (married/cohabited = 1), area of residence (urban = 1 vs. rural = 0).

#### Health

We included 19 indicator variables for each of the following conditions that were obtained from participants' self-report (1 = present, 0 = absent): hypertension, diabetes, hypercholesterolemia, heart disease (angina pectoris, heart failure), embolism, stroke or cerebral thrombosis, arthritis or rheumatism, bronchitis, or pulmonary emphysema, osteoporosis, kidney chronic disease, tuberculosis, cataracts (one or both eyes), urinary incontinence, prostate disease (men only), and cancer (skin/melanoma, cervix, breast, prostate, stomach, leukemia / blood). With this information, we generated a count variable to express the total number of chronic conditions in each participant. This variable was included in our regression models as a proxy for the general health status.

#### Use of Health Services

We measured this variable using two indicators: number of outpatient visits in the last 6 months, and the number of hospitalizations in the last 12 months.

#### Socioeconomic Status (SES)

For SES measurement, we created an asset index following a standard approach proposed by the WHO to estimate the permanent income of the household through the ownership of goods and some characteristics of the housing (28–30). In particular, we used nine dichotomous variables (yes/no) that evaluated possession of household assets. The asset index was created using a polychoric correlation matrix through the application of principal component analysis (PCA). The first component explained 44% of the variation in the data. The index generated by PCA is continuous, with higher values denoting higher household SES.

### Statistical Analysis

Variables were described using means (standard deviations) or proportions, as appropriate. In bivariate analysis, the following statistical procedures were used according to the characteristics of each variable: Chi-squared test for categorical data and Mann-Whitney test for continuous data. Exploratory analyses, including graphs (histograms and scatter plots), were also used to determine the probability distributions of the outcome variable. At this stage, we observed a considerable percentage of OA that did not show out-of-pocket expenses (30%), and the distribution of expenditure showed a right-skewed distribution, so in the multivariate analysis we used a two-part regression model (14, 31).

In the Two-Part model, the outcome variable is analyzed in two phases. In the first part, a regression for dichotomous variables (logit or probit) is used to estimate the probability of incurring OOP health expenditures. In our case, we coded the dependent variable (OOP health expenditures) as a binary indicator, where one equaled older adult that incurred OOP health expenditures and zero equaled those that did not. In the second part, to reduce the effect of skewness of healthcare costs, a linear regression model on the natural logarithm of this variable is applied to estimate the average expenditures among individuals that incurred health expenditures. In general, it is recommended the use of the natural logarithm of healthcare costs to reduce the effects of the skewed nature of this variable. Results are interpreted (β) as semi-elasticities (i.e., as the percentage increase observed in the response variable for one increment in the independent variable). Finally, total expenditures for each individual are estimated by multiplying the probability by the average results obtained through the first and second parts of the model, using Duan's smearing retransformation to obtain fitted values (32).

We further conducted several sensitivity analyses to check the robustness of our results. First, we examined the combined effect of ADL and IADL dependence on OOP health expenditures. The combined effect of ADL and IADL dependence was analyzed by creating a new dichotomous variable, where 1 reflected the simultaneous presence of ADL & IADL, and 0 the presence of neither. Second, instead of modeling mean of healthcare expenditures, we modeled the median of OOP healthcare expenditures using a quantile regression model (33, 34).

All regression models were adjusted for variables described in the Covariates section. Statistical analyses were performed using STATA version 15.1 software (StataCorp. 2015. College Station, TX.). Ninety-five percent confidence intervals and *p*-values were reported. Differences were considered statistically significant if *p* < 0.05.

## Results

The following sociodemographic and health characteristics were observed in our sample. Mean age of participants was 71.1 years (*SD* = 8.4 years). 57.8% were female, 56.0% married/cohabited, and 31% current workers. Mean of years of formal education was 4.2 (*SD* = 4.4). Regarding health characteristics, 63.4% reported at least one chronic condition, and 32.1% had multimorbidity, defined as the presence of two or more chronic diseases. Prevalence of ADL and IADL dependence was 8.6 and 15.0%, respectively.

Table 1 displays the study sample characteristics according to the presence of ADL and IADL dependence. Overall, dependent OA were mostly female (*p* < 0.01), older (*p* < 0.01), and single or widowed (*p* < 0.01). They worked less (*p* < 0.01) and had fewer years of schooling (*p* < 0.01). They had more visits to a physician (*p* < 0.01), more hospital overnight stays (*p* < 0.01), and a greater number of chronic diseases (*p* < 0.01). Regarding OOP healthcare expenditures, dependent OA had a greater probability of incurring expenses than non-dependent OAs (*p* < 0.01) and also a higher annual mean expenditure (*p* < 0.01). This general profile was similar for ADL and IADL dependent OA. Finally, OA with ADL dependence had an unadjusted total annualized mean OOP healthcare expenditure of $29,272 (Mexican pesos), while the amount in OAs with IADL was $18,196.

**Table 1:** Health and sociodemographic characteristics of older adults according to the status of ADL and IADL dependence^a^.

	**ADL^b^ dependence**		***p*-value**	**IADL^c^ dependence**		***p*-value**
	**No**	**Yes**		**No**	**Yes**	
	***n* = 672**	***n* = 63**		***n* = 625**	***n* = 110**	
	**(91.4%)**	**(8.6%)**		**(85.0%)**	**(15.0%)**	
**Outcomes**						
Probability of incurring OOP^d^ healthcare expenditures	0.63	0.84	<0.01	0.62	0.75	<0.01
Mean annual OOP healthcare expenditures	7,532 (27,135)	29,272 (61,925)	<0.01	7,847 (28,192)	18,197 (48,187)	<0.01
**Covariates**						
Sex (female = 1)	55.8	79.4	<0.01	55.3	71.9	<0.01
Age	70.3 (7.7)	79.5 (10.4)	<0.01	69.7 (7.5)	79.0 (9.2)	<0.01
Marital status (married/civil union = 1)	58.5	30.2	<0.01	58.8	40.0	<0.01
Years of formal schooling	4.3 (4.4)	2.8 (3.2)	<0.01	4.4 (4.5)	2.6 (2.9)	<0.01
Health coverage (yes = 1)	89.0	85.7	0.43	88.3	90.9	0.43
Number of chronic diseases	1.2 (1.3)	2.1 (1.6)	<0.01	1.2 (1.3)	1.8 (1.6)	<0.01
Retirement pension (yes = 1)	34.2	39.7	0.38	32.3	48.2	<0.01
Labor status (current worker = 1)	33.5	4.8	<0.01	36.2	1.8	<0.01
Socioeconomic status (asset index)	0.1 (2.0)	0.3 (1.9)	0.52	0.1 (2.0)	0.4 (1.9)	0.08
Dwelling area (urban = 1)	75.4	82.5	0.21	75.4	80.0	0.29
Number of visits to physician in the last 6 months	1.7 (2.8)	2.8 (3.2)	<0.01	1.7 (2.8)	2.5 (2.9)	<0.01
Number of hospital overnight stays in the last 12 months	0.2 (1.8)	3.0 (12.2)	<0.01	0.2 (1.8)	1.8 (9.3)	<0.01

a *Cells are percentages, means (SD), or proportions*.

b *ADL, basic activities of daily life*.

c *IADL, instrumental activities of daily life*.

d *OOP, Out-of-pocket*.

The results of OOP healthcare expenditures for specific items are shown in Tables 2, 3. For ADL dependence, expenditures from medications, nursing services, and medical consultations, mostly contributed to total OOP healthcare expenditures (Table 2). As for IADL dependence, medications and nursing services were the highest contributors (Table 3).

**Table 2:** Out-of-pocket health expenditures by specific cost items according to the status of ADL dependence.

	**Probability of incurring out-of-pocket healthcare expenditures^a^**			**Annual mean out-of-pocket healthcare expenditures**		
	**ADL^b^ dependence**			**ADL dependence^c^**		
	**No**	**Yes**	***p*-value**	**No**	**Yes**	***p*-value**
**DIRECT COSTS**						
**Medical**						
Medical consultations	0.21	0.33	0.03	1,425 (7,210)	4,000 (9,252)	<0.01
Medical specialist consultations	0.09	0.14	0.15	303 (2,890)	676 (2,749)	0.13
Medicines (including vitamins)	0.36	0.59	<0.01	4,199 (16,897)	13,782 (31,999)	<0.01
Laboratory tests	0.08	0.11	0.32	413 (2,374)	457 (2,541)	0.36
Hospitalization	0.02	0.17	<0.01	305 (2,945)	1,738 (6,856)	<0.01
**Non-medical**						
Nursing services	0.01	0.08	<0.01	49 (626)	5,035 (30,626)	<0.01
Devices (visual, hearing, orthopedic) or prosthesis	0.13	0.19	0.15	325 (2,254)	124 (368)	0.17
Transportation to health units	0.22	0.41	<0.01	296 (1,383)	1,425 (4,063)	<0.01
Others (personal hygiene, healing material, traditional medicine)	0.18	0.36	<0.01	188 (1,375)	1,377 (3,267)	<0.01
**INDIRECT COSTS**						
**Morbidity**						
Loss labor productivity (older adult)	0.01	0.13	<0.01	22 (308)	429 (2,541)	<0.01
Loss labor productivity in the household (caregiver or relative)	0.01	0.17	<0.01	8 (84)	229 (827)	<0.01

a *Cells are proportions*.

b *ADL, basic activities of daily life*.

c *Means and standard deviations*.

**Table 3:** Out-of-pocket health expenditures by specific cost items according to the status of IADL dependence.

	**Probability of incurring out-of-pocket healthcare expenditures^a^**			**Annual mean out-of-pocket healthcare expenditures**		
	**IADL^b^ dependence**			**IADL dependence^c^**		
	**No**	**Yes**	***p*-value**	**No**	**Yes**	***p*-value**
**DIRECT COSTS**						
**Medical**						
Medical consultations	0.21	0.29	0.06	1,517 (7,514)	2,375 (6,953)	<0.01
Medical specialist consultations	0.09	0.12	0.31	320 (2,995)	424 (2,106)	0.13
Medicines (including vitamins)	0.36	0.50	<0.01	4,464 (17,626)	8,177 (24,460)	<0.01
Laboratory tests	0.08	0.07	0.79	439 (2,457)	289 (1,947)	0.36
Hospitalization	0.03	0.11	<0.01	241 (2,167)	1,486 (7,281)	<0.01
**Non-medical**						
Nursing services	0.01	0.05	<0.01	40 (566)	2,956 (23,237)	<0.01
Devices (visual, hearing, orthopedic) or prosthesis	0.12	0.17	0.17	344 (2,336)	102 (315)	0.17
Transportation to health units	0.21	0.35	<0.01	258 (1,163)	1,162 (3,658)	<0.01
Others (personal hygiene, healing material, traditional medicine)	0.19	0.26	0.06	197 (1,424)	816 (2,554)	<0.01
**INDIRECT COSTS**						
**Morbidity**						
Loss labor productivity (older adult)	0.01	0.07	<0.01	24 (320)	245 (1,928)	<0.01
Loss labor productivity in the household (caregiver or relative)	0.01	0.14	<0.01	2 (38)	165 (654)	<0.01

a *Cells are proportions*.

b *IADL, instrumental activities of daily life*.

c *Means and standard deviations*.

The results of the adjusted two-part regression models are presented in Table 4. Neither ADL dependence (OR = 1.86, CI95%: 0.85–4.11) nor IADL dependence (OR = 1.27, CI95%: 0.72–2.25) was found to be associated with the probability of incurring total annual OOP expenditures. We also did not observe a significant association for the combined presence of ADL and IADL dependence (OR = 1.73, CI95%: 0.78–3.81).

**Table 4:** Results of two-part and quantile regression models^*^.

	**Two-Part regression model**						**Quantile regression**		
	**Probability of incurring out-of-pocket healthcare expenditures**			**Annual mean out-of-pocket healthcare expenditures**			**Annual median out-of-pocket healthcare expenditures**		
	**Logistic model**			**Linear model (log y)**			
	**OR^c^**	**CI^d^ 95%**		**β^e^**	**CI 95%**		**Coefficient**	**CI 95%**	
ADL^a^ dependence	1.86	0.85	4.11	1.07	0.43	1.71	5789.6	3861.9	7717.4
IADL^b^ dependence	1.27	0.72	2.25	0.97	0.49	1.45	1844.4	267.4	3421.3
ADL and IADL combined	1.73	0.78	3.81	1.32	0.74	1.90	8178.0	6252.8	10103.2

* *All models were adjusted for covariates showed in Table 1*.

a *ADL, basic activities of daily life*.

b *IADL, instrumental activities of daily life*.

c *OR, odds ratio*.

d *CI, confidence interval*.

e *β, Beta Coefficient*.

The presence of ADL dependence was associated with an 107% higher mean OOP health expenditure compared to non-dependent OA (β = 1.07, CI95%: 0.43–1.71). This was 97% for IADL dependence (β = 0.97, CI95%: 0.49–1.45). The combined effect of ADL and IADL dependence (132%) was greater (β = 1.32, CI95%: 0.74–1.90) than the isolated presence of ADL or IADL dependence.

With regard to the median of OOP healthcare expenditure, we found that ADL dependent individuals had a median annual expenditure $5,798 higher than non-dependent individuals (Mexican pesos), while the IADL dependent OA had an expenditure that was $1,844 higher than their non-dependent counterparts. This number was greater for the combined ADL and IADL dependence ($8,178 Mexican pesos) (Table 4).

**Figure 1:**
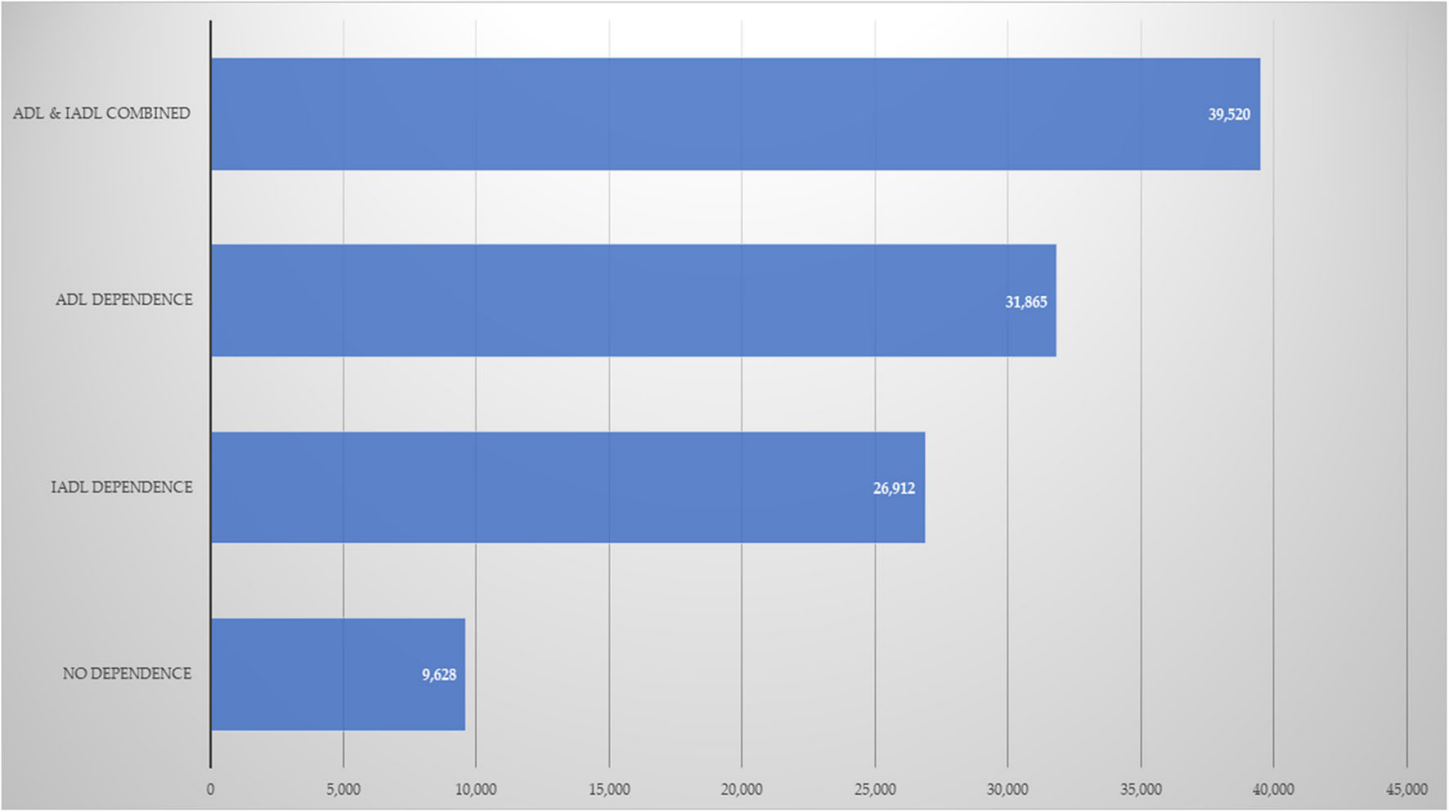
Annual mean OOP healthcare expenditures (Mexican pesos). ADL, basic activities of daily life; IADL, instrumental activities of daily life.

**Figure 2:**
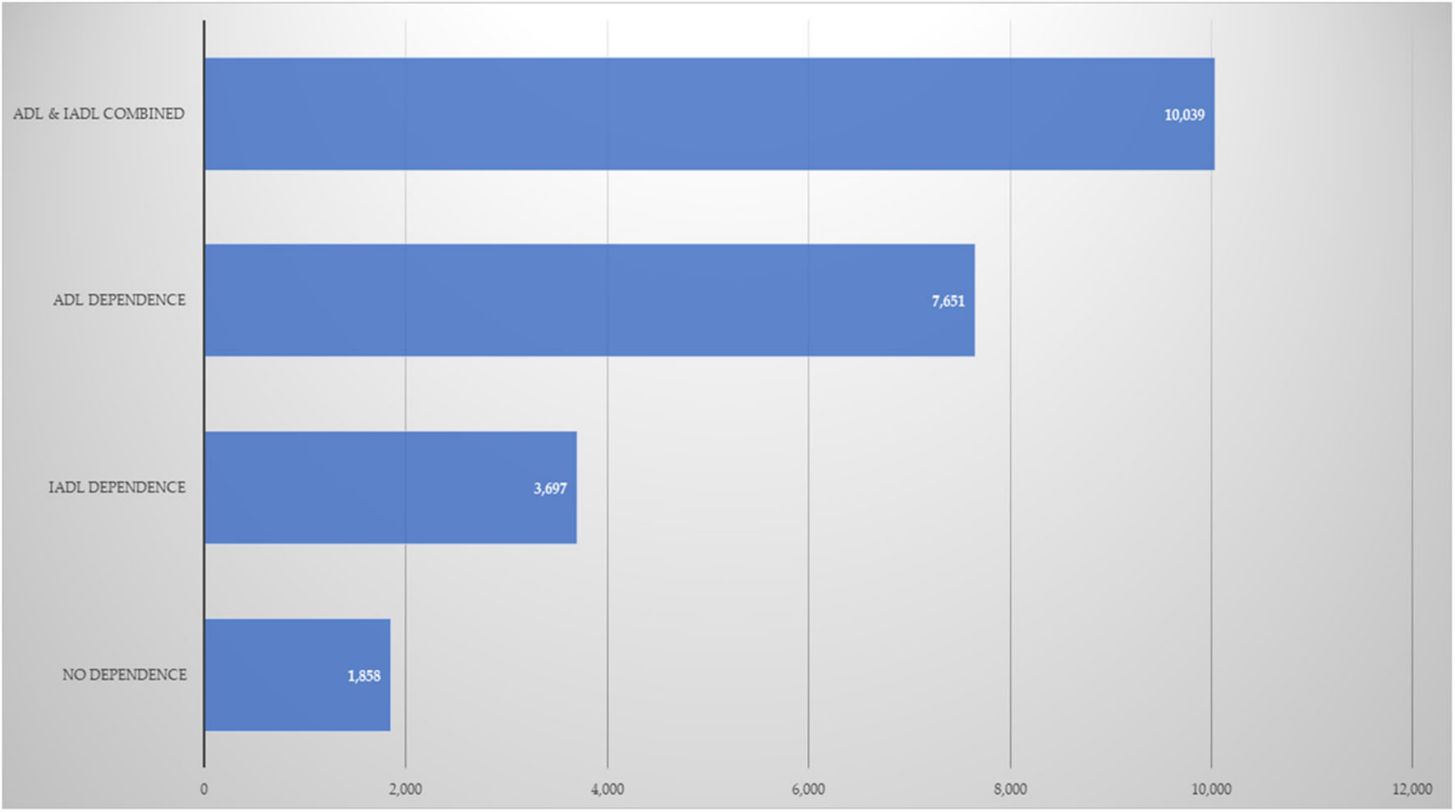
Annual median OOP healthcare expenditures (Mexican pesos). ADL, basic activities of daily life; IADL, instrumental activities of daily life.

Finally, Figures 1, 2 show the estimated OOP healthcare expenditures according to the presence of dependence in OA. Figure 1 displays the data for mean annual expenditure, while Figure 2 displays median expenditure. The average expenditure was 4.1, 3.3, and 2.8 times larger for the ADL and IADL, ADL-only, and IADL-only groups, in relation to the group without dependence. Meanwhile, the same data for median expenditure was 5.4, 4.1, and 1.9, respectively.

## Discussion

Our findings corroborate that there is a significant association between dependence in OA and OOP healthcare expenditures. Based on a sample of 735 community-dwelling older Mexican adults, we observed that the presence of ADL or IADL dependence as well as their combined presence was associated with a significant increase in the total annualized OOP healthcare expenditures.

Consistent with previous studies, we found a significant association between dependence in OA and healthcare costs. One study with European older adults age 65+ found that ADL and IADL dependence were associated with higher societal costs (including primary, secondary, social, home, and informal care). Although, this study measured ADL and IADL dependence on a continuous scale and was focused on societal costs rather than OOP expenditures (17). Even so, we similarly found that dependent OA tend to have higher healthcare cost than their independent counterparts. Another study among US adults found that ADL and IADL difficulties were associated with total annual cost of care (TACC). In this study, however, ordinal variables were used to measure ADL and IADL difficulties (0, 1–2, and 3 or more difficulties), and results showed that having three or more difficulties was associated with a higher TACC (18). In our study, we observed significant associations from the presence of a single difficulty. Two possible explanations could be advanced for this difference. First, we just include OA while they included adults age 18 or more, so our study sample could have worse health and functional conditions that would make it possible to observe significant associations with minor levels of ADL or IADL difficulties. Second, our definition of dependence includes not just difficulties in ADL or IADL, but also the need for someone to help in the execution of these activities. Finally, a study conducted with older US adults, including beneficiaries of Medicare and participants of the Health and Retirement Study, reported that compared to those with no ADL impairment, the most severely impaired (two or more ADL difficulties) had a 33% higher cost (19). It is interesting to note that the sample considered for this study were OA who were admitted to a hospital at least once in a previous period of 10 years, while our sample was comprised of community-dwelling individuals who were not hospitalized or institutionalized. Despite this difference, our results are consistent regarding the association between ADL dependence and OOP healthcare costs.

Despite the similarity in the described results, we should note that none of the studies -to the best of our knowledge- on the economic impact of dependency have used the two-part model approach. This fact has two important implications. On the one hand, the results are not strictly comparable, given that those studies conceive of healthcare spending as a single-stage process (i.e., the estimation of the total amount disbursed). Meanwhile, the two-part model recognizes the potential presence of two phases: (a) to incur or not in healthcare expenditures (i.e., the probability of incurring in spending), and (b) given the incidence of healthcare expenses, what was the amount disbursed? (i.e., the estimation of the average expenses).

On the other hand, we found that neither ADL nor IADL was associated with the probability of incurring OOP healthcare expenditures (even the two conditions combined). Although a similar result has not been reported in the literature, one study found that the severity of disability in older adults, assessed through the WHO Disability Assessment Schedule -WHODAS 2.0, was associated with a higher probability of healthcare expenses (14). Given that disability reported in that study included six domains (cognition, mobility, self-care, getting along, life activities, and social participation) that encompassing more characteristics than a single component of physical limitations, then the isolated presence of physical dependency could not be enough to increase the probability of having healthcare expenses. However, future studies are needed to confirm or dismiss this hypothesis.

The results of our study have several implications. First, the high prevalence of dependence among OA calls for concerted efforts of healthcare professionals, clinical researchers, and policy-makers to design effective interventions that prevent or ameliorate the adverse effects of dependence in advanced ages. For example, in Mexico the creation of a National Care System (*Sistema Nacional de Cuidados*, SNC by its Spanish acronym) is currently being considered. The objective would be to provide public services that are accessible, pertinent, of quality, and sufficient to guarantee the right of all people to be cared for, and also the rights of caregivers (35). It is expected to be financed with public funds, but also through the families' corresponsability. In particular, the SNC proposes that a catalog of care services be generated that includes financial benefits, home care assistance and care support centers. It would also include support for unpaid carers through a cash payment (for hiring a caregiver). Second, the health systems should be prepared for the increased demand on the use of health services by dependent OA, given that the OOP healthcare expenditures of these individuals will grow exponentially in the next few years. Third, gerontological research on the functional status of the older adults should highlight the joint assessment of ADL and IADL dependence for the estimation of healthcare expenditures, because their economic impact might be more significant than either on its own.

Our study has some strengths. To the best of our knowledge, our study is the first designed to deliberately estimate the economic impact of ADL and IADL dependence. It is also the first attempt to analyze the combined effects of ADL and IADL dependence on OOP healthcare expenditures. Nevertheless, some limitations must be considered also. First, recall and survivor bias can be limitations for epidemiological studies with older adult participants. It could be, for example, that older adults with chronic conditions tend to overestimate their healthcare expenditures in comparison with healthiest older adults. Second, healthcare costs variables are self-reported. This may have led to overestimation or underestimation of true OOP healthcare expenditures. In spite of this, consistent associations between dependence and healthcare costs have been reported previously in the literature (16, 19). Third, the results from the current study came from a cross-sectional study, and thus a causal pathway between dependence and OOP healthcare expenditures cannot be determined. And fourth, our measurement of dependency has been expressed dichotomously, which implies that severity levels of dependency are not properly captured, which could restrict the generalization of our results to even more vulnerable populations, like OA with a greater number of limitations in ADL or IADL.

## Conclusion

Our study shows that ADL and IADL dependence are associated with total annualized OOP healthcare expenditures. This association is even higher when both conditions are present together. These findings tried to highlight the economic implications of dependence on individuals, their families, and the health system. Finally, given that the current evidence is insufficient to conclude which strategies to prevent dependence in OA are more effective, future studies should be conducted to estimate the costs and effectiveness of different interventions to address dependence in OA.

## Data Availability Statement

The datasets analyzed during the current study are freely available in the figshare repository [doi: 10.6084/m9.figshare.9978599].

## Ethics Statement

The ethics and Research Committees of the National Institute of Public Health in Mexico approved the study economic impact of physical dependence in older adults and the burden of informal care (No. 1203-2018). All participants were provided with a detailed explanation of study procedures and signed an informed consent letter.

## Author Contributions

AS-R, BM-E, and JM-H conceived and designed the study. AS-R and JM-H carried out the analysis. AS-R, IT, and JM-H drafted the manuscript. All authors reviewed, edited, contributed to rewriting of the manuscript, and granted final approval to the version submitted for publication.

## Conflict of Interest

The authors declare that the research was conducted in the absence of any commercial or financial relationships that could be construed as a potential conflict of interest.
